# The significance of serum S100 calcium-binding protein A4 in silicosis

**DOI:** 10.1186/s12890-022-01918-y

**Published:** 2022-04-04

**Authors:** Jing Zhang, Cuifang Yuan, Enhong Li, Yiming Guo, Jie Cui, Heliang Liu, Xiaohui Hao, Lingli Guo

**Affiliations:** School of Public Health, Hebei Key Laboratory for Organ Fibrosis, North China University of Science and Technology, Tangshan, 063210 Hebei China

**Keywords:** Silicosis, S100 calcium-binding protein A4, Fibrosis, Inflammation

## Abstract

**Background:**

Silicosis is a chronic occupational pulmonary disease characterized by persistent inflammation and irreversible fibrosis. Considerable evidences now indicate that S100 calcium-binding protein A4 (S100A4) has been associated with fibrotic diseases. However, the role of S100A4 in silicosis is still unclear.

**Methods:**

In this study, serum levels of S100A4, transforming growth factor-β1 (TGF-β1), connective tissue growth factor (CTGF), interleukin-6 (IL-6) and tumour necrosis factor-α (TNF-α) in patients with silicosis (n = 42) and control group (CG, n = 12) were measured by ELISA. S100A4 expression in lung tissues and primary alveolar macrophages (AMs) of mice with and without silicosis was detected by immunohistochemistry (IHC)/real-time PCR. The correlations between S100A4 and cytokines or lung function were assessed by Spearman's rank correlation analyses.

**Results:**

Compared with CG, the levels of S100A4 were significantly increased in silicosis patients (70.84 (46.22, 102.46) ng/ml vs (49.84 (42.86, 60.02) ng/ml). The secretions of TGF-β1, CTGF, IL-6 and TNF-α in silicosis group were significantly higher than that in control group (*p* < 0.05). Serum S100A4 levels were positively correlated with TGF-β1 and IL-6, while were negatively correlated with lung function parameters including percentage of predicted forced vital capacity (FVC%pre), maximum vital capacity (Vcmax), deep inspiratory capacity (IC) and peak expiratory flow at 75% of vital capacity (PEF75). In receiver operating characteristic (ROC) analyses, S100A4 > 61.7 ng/ml had 63.4% sensitivity and 83.3% specificity for silicosis, and the area under the curve (AUC) was 0.707. Furthermore, immunostaining of lung tissues showed the accumulation of S100A4-positive cells in the areas of nodules of silicotic mice. The mRNA expression of S100A4 in the lung tissues and AMs of silicotic mice were significantly higher than controls.

**Conclusion:**

These data suggested that increased S100A4 might contribute to the pathogenesis of silicosis.

## Introduction

Silicosis is one of the most common occupational diseases caused by the chronic inhalation of large amounts of respirable crystalline silica from the environment [1, 2]. Characteristic pathology in silicosis consists of persistent inflammation, formation of silicotic nodules, central hyalinization and excessive deposition of extracellular matrix, which lead to lung function insufficiency [1, 3]. The underlying cause of silicosis is not well known and there has been little progress in therapies.

When crystalline silica are inhaled and then engulfed by alveolar macrophages, many pro-inflammatory and pro-fibrotic cytokines, such as transforming growth factor-β1 (TGF-β1) [4], connective tissue growth factor (CTGF) [5], tumour necrosis factor-α (TNF-α) [6] and interleukin-6 (IL-6) [7], are released to trigger inflammatory cell infiltration, followed by fibroblast proliferation and collagen deposition [8]. These findings suggest critical roles of TGF-β1, CTGF, TNF-α and IL-6 in silicosis. It has been found that TGF-β1-stimulated endometrial cancer cell [9] and TNF-α-stimulated airway smooth muscle tissues [10] present an increase in S100 calcium-binding protein A4 (S100A4) protein. The expression of CTGF was positively correlated with S100A4 in samples of primary human breast cancer [11]. And treatment of peripheral blood mononuclear cells (PBMCs) with S100A4 significantly induced the synthesis of TNF-α and IL-6 [12]. Theses researches suggest a possible crosstalk between S100A4 and TGF-β1, CTGF, TNF-α and IL-6.

S100A4 is a member of the S100 proteins containing two calcium-binding motifs. S100A4 is well known to not only promote cell motility, invasion and autophagy [13–15], but also involves in the inflammatory and fibrotic processes [16, 17]. Furthermore, increasing evidences indicate that S100A4 participate in the process of pulmonary fibrosis [18–20]. It has been found that S100A4 secreted by alveolar macrophages contributes to fibrosis by promoting the proliferation and activation of lung fibroblasts [19]. And upregulation of S100A4 was observed in the serum and bronchoalveolar lavage fluid (BALF) of idiopathic pulmonary fibrosis (IPF) patients [18, 21]. However, the relationship between S100A4 and silicosis is still unknown. This study aims to investigate the possible role of S100A4 in silicosis.

## Materials and methods

### Study subjects

In this study, 42 silicosis cases were enrolled from Beidaihe Chinese coal workers nursing home and 12 control subjects were enrolled from an iron mine of Henan province. The diagnosis criteria on silicosis were based on clinical and radiological findings on high quality X-ray according to diagnostic criteria of pneumoconiosis (GBZ70-2015, China). The criteria define silicosis as stages I, II and III, corresponding to mild, moderate and severe respectively. Specifically, the lung field is divided into mutually exclusive six subregions: left-top, left-middle, left-bottom, right-top, right -middle, right-bottom. The profusion levels of small opacities in the lung are described in level 1, 2, 3. Stage I is defined as: level 1 profusion of small opacities presented in more than two subregions. Stage II is defined as: level 2 or 3 profusion of small opacities presented in four subregions or more. Stage III is defined as: large opacities presented [22]. Exclusion criteria for this study were as follows: subjects with other inflammatory diseases, other fibrotic diseases, other pulmonary diseases, such as chronic obstructive pulmonary disease (COPD), active tuberculosis, pneumonia and pulmonary heart disease and autoimmune disorders. Data of lung function were collected by Puritan Bennett™ 840 Ventilator [23]. All subjects gave their informed consent for inclusion before they participated in the study. The study was conducted in accordance with the Declaration of Helsinki, and the protocol was approved by the Clinical Trial and Ethics Committee of North China University of Science and Technology (approval number 16028).

### Measurement for S100A4, TGF-β1, CTGF, TNF-α and IL-6

Peripheral blood samples were obtained from both silicosis patients and control group (CG). The serum was separated at 3000 rpm for 10–15 min and subsequently frozen in a − 80 °C freezer until analysis. Serum S100A4 was measured using human S100A4 ELISA kit from Cusabio (Wuhan, China); TGF-β1, CTGF and IL-6 were measured using ELISA kits from BOSTER (Wuhan, China); TNF-α level in serum was measured by ELISA assay from eBioscience (San Diego, California).

### Animals treatment and alveolar macrophages extraction

12 specific pathogen-free, male C57BL/6 mice weighing 18–20 g were purchased from Beijing Huafukang Bioscience Co., Inc. (Beijing, China) and subsequently bred under SPF conditions with controlled room temperature (22–25° C) and a 12-h light/dark cycle. They were randomly divided into 2 groups. In silica group, mice were anesthetized by inhalation with 4% isoflurane in O_2_, and then received a single-dose intranasal instillation of 5 mg crystalline silica oxide powder (SiO_2_; Sigma Chemical Co, St. Louis, MO; particle size 0.5–10 mm) diluted in 50 μl sterile saline (0.9% NaCl) (n = 6) [24], while the same volume of sterile saline was instilled in the control group (n = 6).

Mice were intraperitoneally anesthetized with 50 mg/kg sodium pentobarbital at 14d. The lungs were lavaged by eight 1-ml boluses of sterile saline. And the recovered saline was centrifuged at 1000 rpm for 10 min. The cell pellet was resuspended in warm DMEM supplemented with 10% fetal bovine serum, 0.1 mg/mL streptomycin and 100U/mL penicillin. Alveolar macrophages (AMs) were purified by removing non-adherent cells following culture for 1 h.

The experiment was approved by the Animal Care and Use Committees of North China University of Science and Technology (approval number 2016037), and followed the guidelines for animal care and use. This study was reported in accordance with ARRIVE guidelines.

### Immunohistochemistry

The lung tissues of mice were fixed in 4% formaldehyde (pH 7.4) and embedded in paraffin. Fixed-tissues were cut into 4-µm-thick slices. Then the slices were incubated with a primary antibody against S100A4 (1:100, Zenbio, Chengdu, China) overnight at 4 °C, followed by an incubation with secondary antibody for 1 h at room temperature (ZhongshanJinqiao Biotechnology, Beijing, China). The slices were scanned by microscope (Olympus, Tokyo, Japan) to view the images.

### Isolation of RNA and real-time PCR

The total RNA were isolated from lung homogenates and AMs of mice using TRIzol Reagent (Invitrogen, Carlsbad, CA, USA), according to the manufacturer’s protocol. cDNA was then synthesized using the PrimeScript RT reagent kit (Takara Bio Inc., Kyoto, Japan), and then PCR amplification was performed in triplicate using TB Green® Premix Ex Taq™ II kit (Takara Bio Inc., Kyoto, Japan). Primers for S100A4 and β-actin were as follows: S100A4 (forward) 5′-TGTCCACCTTCCACAAATACTCAG-3′ and (reverse) 5′-GTTGCTGTCCAAGTTGCTCATC-3′, β-actin (forward) 5′-CTAAGGCCAACCGTGAAAG-3′ and (reverse) 5′-ACCAGAGGCATACAGGGACA-3′. Data were normalized to the expression of β-actin, and relative expression levels were determined using the 2^−ΔΔCt^ method.

### Statistical analysis

Data analysis was conducted using SPSS 21 for Windows. Normally distributed data were presented as the mean ± standard deviation and compared using ANOVA analysis or Student’s t-test. Nonparametric data were expressed as median (25th, 75th percentile) and were analysed using Kruskal–Wallis H test or Mann–Whitney U test. Categorical variables were analysed with a Chi-square test. Spearman's rank correlation coefficients were performed to determine associations between S100A4 and cytokines or lung function in silicosis. A *p* value of < 0.05 was considered to be statistically significant. Receiver operating characteristic (ROC) curve analysis was used to test the role of S100A4 in the discrimination for silicosis.

## Results

### Clinical characteristics of subjects

The characteristics of subjects are shown in Table 1. There were no significant differences in sex ratio, age, smoking pack-year, years of occupational exposure to silica dust and body mass index (BMI) between CG and silicosis group. Compared with CG, the lung function parameter percentage of predicted forced vital capacity (FVC%pre), percentage of predicted forced expiratory volume in one second (FEV1%pre) and ratio of forced expiratory volume in one second to forced vital capacity (FEV1/FVC) were significantly decreased in patients with silicosis (*p* < 0.05), while other parameters did not differ between groups. The silicosis patients were divided into stage I, II, III, and the proportion of three subgroups were 21.43%, 30.95% and 47.62% respectively.

**Table 1:** Clinical characteristics of study subjects

	CG (n = 12)	Silicosis (n = 42)	*p*-value
Male/female	10/2	41/1	0.234
Age (yrs)	40.92 ± 11.49	46.60 ± 5.95	0.063
Smoking pack-years (yrs)	15.00 (2.50, 20.00)	13.50 (4.25, 23.75)	0.742
Years of occupational exposure to silica dust (yrs)	9.56 ± 10.07	15.26 ± 7.75	0.114
BMI	24.19 (20.18, 26.64)	21.76 (20.05, 23.84)	0.213
FVC (l)	3.38 ± 0.52	3.24 ± 0.74	0.725
FVC% pre	87.30 ± 8.16	71.51 ± 14.27	**0.037**
FEV1 (l)	2.73 ± 0.45	2.33 ± 0.79	0.333
FEV1% pre	85.45 (72.98, 87.20)	60.30 (48.55, 72.98)	**0.021**
FEV1/FVC (%)	80.22 ± 1.10	69.96 ± 14.21	** < 0.01**
Vcmax (l)	3.42 ± 0.54	3.26 ± 0.68	0.642
IC (l)	2.01 ± 0.05	2.09 ± 0.69	0.485
PEF (l/s)	3.88 (2.91, 3.90)	2.95 (2.35, 4.29)	0.416
PEF25 (l/s)	3.88 (2.82, 3.90)	2.81 (2.04, 3.69)	0.214
PEF50 (l/s)	3.48 (2.49, 3.61)	1.98 (1.29, 3.18)	0.123
PEF75 (l/s)	1.42 (1.23, 1.70)	0.97 (0.52, 1.44)	0.072
*Stage of silicosis (n, %)*			–
Stage I	–	9 (21.43%)	
Stage II	–	13 (30.95%)	
Stage III	–	20 (47.62%)	

The value of* P* was in bold indicating* P* < 0.05

BMI, body mass index; FVC, forced volume capacity; FEV1, forced expiratory volume in 1 s; FVC% pre, percentage of predicted forced vital capacity; FEV1/FVC, ratio of forced expiratory volume in one second to forced vital capacity; FEV1% pre, percentage of predicted forced expiratory volume in 1 s; Vcmax, maximum vital capacity; IC, deep inspiratory capacity; PEF, peak expiratory flow; PEF25, peak expiratory flow at 25% of vital capacity; PEF50, peak expiratory flow at 50% of vital capacity; PEF75, peak expiratory flow at 75% of vital capacity

### Serum levels of S100A4, TGF-β1, CTGF, IL-6 and TNF-α

**Fig. 1:**
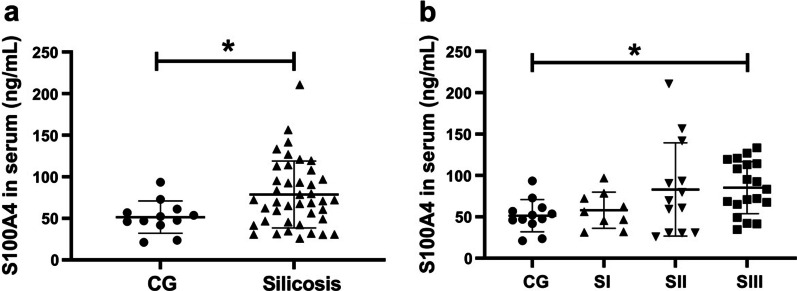
Serum levels of S100A4 in subjects. **a**, **b** The secretion of serum S100A4 in control group and silicosis patients was detected by ELISA. CG, control group; SI, stage I of silicosis; SII, stage II of silicosis; SIII, stage III of silicosis. *p < 0.05

To investigate the possible role of S100A4 in silicosis, we first detected the secretion of S100A4, inflammatory indicator IL-6 and TNF-α, and fibrotic cytokine TGF-β1 and CTGF in the serum of patients with silicosis. As shown in Fig. 1a, serum S100A4 were significantly increased in silicosis patients (70.84 (46.22, 102.46) ng/ml) compared with control subjects (49.84 (42.86, 60.02) ng/ml). In addition, the levels of S100A4 in SIII (85.23 ± 31.44 ng/ml) were higher than those in CG (51.44 ± 19.50 ng/ml) (Fig. 1b). The secretion of S100A4 in SI and SII were also higher than that in CG, while no statistical difference between groups was found (58.06 ± 21.91 ng/ml, 83.12 ± 56.33 ng/ml vs 51.44 ± 19.50 ng/ml respectively).

**Fig. 2:**
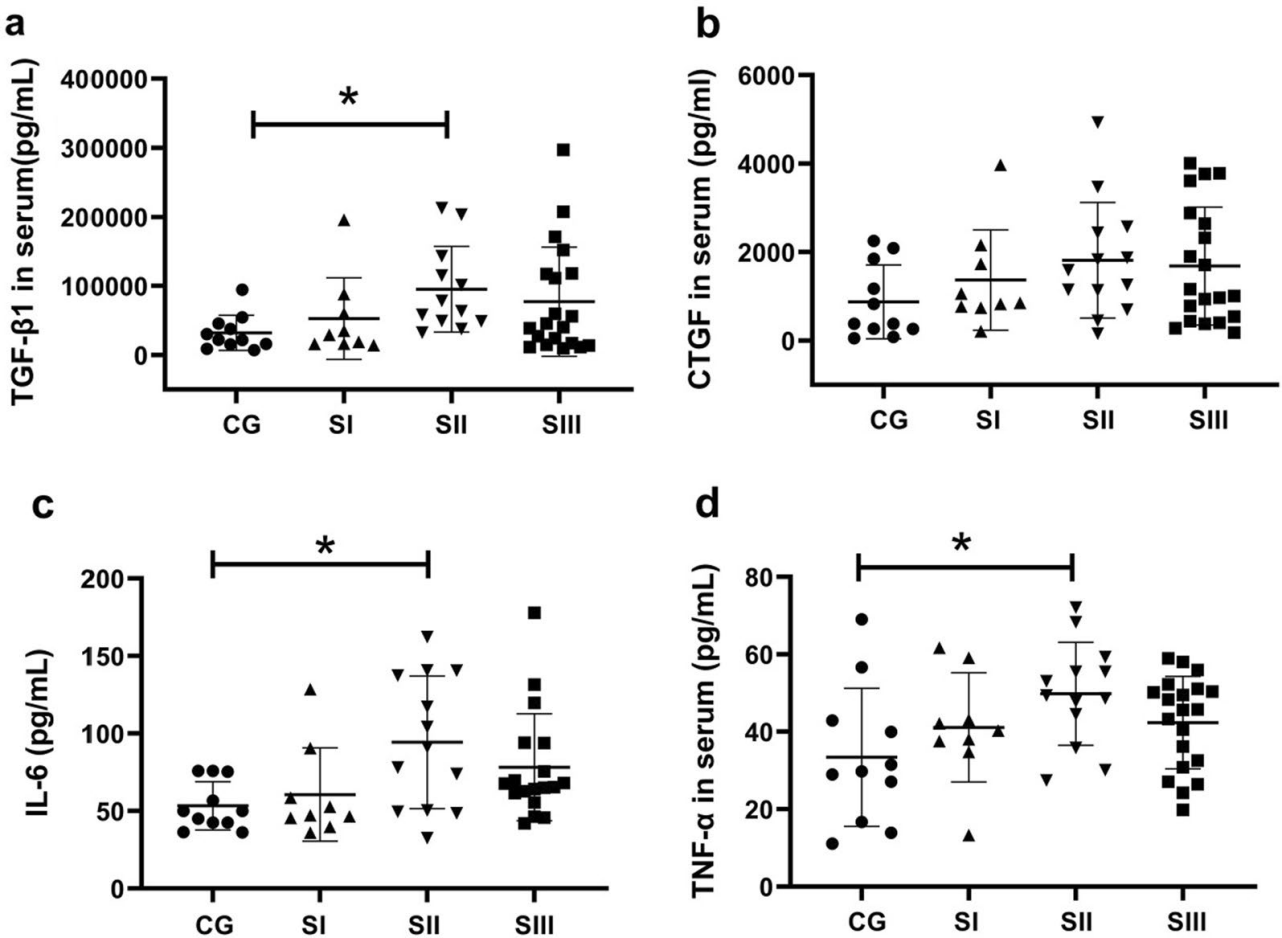
Serum levels of TGF-β1, CTGF, IL-6 and TNF-α in the subjects. The secretions of **a** TGF-β1, **b** CTGF, **c** IL-6 and **d** TNF-α in serum were detected by ELISA. CG, control group; SI, stage I of silicosis; SII, stage II of silicosis; SIII, stage III of silicosis. *p < 0.05

Compared with CG group, the secretions of serum TGF-β1, CTGF, TNF-α and IL-6 were significantly increased in the silicosis group (Table 2). Further investigation found that the concentrations of TGF-β1 in SII group (70,897.18 (48,779.05, 136,059.3) pg/ml) were higher than those in CG group (21,968.10 (15,404.13, 45,419.29) pg/ml) (Fig. 2a). In addition, the levels of IL-6 in SII group (91.27 (49.95, 138.98) pg/ml) were higher than those in CG group (50.10 (42.52, 75.34) pg/ml) (Fig. 2c). Compared with CG group (33.41 ± 17.81 pg/ml), the secretion of TNF-α in SII group was significantly increased (49.81 ± 13.30 pg/ml) (Fig. 2d). Although the secretion of CTGF in SI, SII and SIII group were higher than in CG group, there was no significant difference between groups (Fig. 2b).

**Table 2:** The secretion of cytokines (pg/ml)

	CG (n = 12)	Silicosis (n = 42)	*p*-value
TGF-β1	21,968.10 (15,404.13–45,419.29)	49,046.82 (21,500.08–116,110.40)	**0.027**
CTGF	1001.2 (157.9–2028.7)	1157.3 (730.5–2386.0)	**0.04**
TNF-α	33.41 ± 17.81	44.39 ± 13.04	**0.026**
IL-6	50.10 (42.52–75.34)	66.60 (48.88–101.68)	**0.031**

The value of* P* was in bold indicating* P* < 0.05

### Correlations between S100A4 and cytokines or lung function

Next, the correlations between S100A4 and cytokines or lung function were analyzed. Serum S100A4 had a significant positive association with TGF-β1, IL-6 and had a significant negative association with FVC%pre, Vcmax, IC and PEF75. However, no correlation between serum levels of S100A4 and CTGF, TNF-α or other pulmonary function parameters was found (Table 3).

**Table 3:** The correlations between S100A4 and cytokines and lung function

	S100A4	
	r	*p*-value
TGF-β1	0.334	**0.017**
CTGF	0.272	0.051
TNF-α	− 0.083	0.557
IL-6	0.334	**0.017**
FVC (l)	− 0.187	0.241
FVC% pre	− 0.310	**0.048**
FEV1(l)	− 0.152	0.344
FEV1% pre	− 0.221	0.165
FEV1/FVC (%)	− 0.127	0.430
Vcmax (l)	− 0.361	**0.020**
IC (l)	− 0.328	**0.037**
PEF (l/sec)	− 0.086	0.591
PEF25 (l/sec)	− 0.115	0.473
PEF50 (l/sec)	− 0.208	0.192
PEF75 (l/sec)	− 0.353	**0.023**

The value of* P* was in bold indicating* P* < 0.05

### The cut-off point of S100A4 determined by ROC analysis

**Fig. 3:**
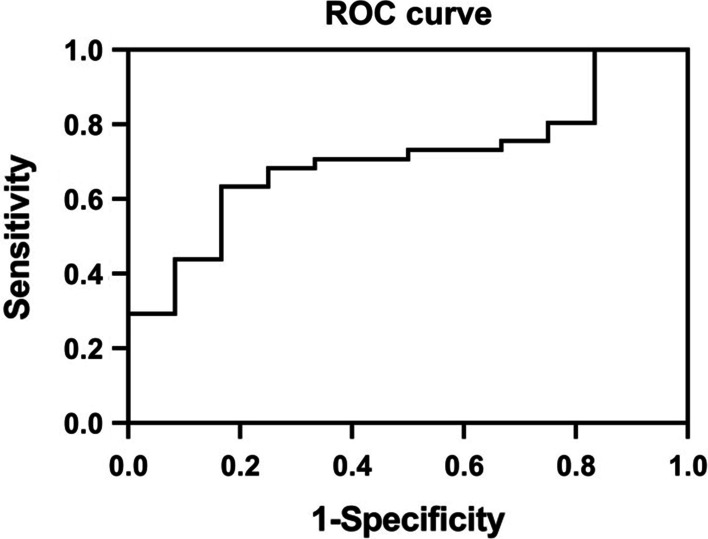
ROC curve for S100A4 (AUC = 0.707) to discriminate between silicosis patients and control group

ROC curve analysis was used to identify the discriminatory power of S100A4 in serum for silicosis. We found that 61.7 ng/ml was the cut-off value of serum S100A4 for detecting silicosis. The sensitivity and specificity were 63.4% and 83.3%, respectively. The area under the curve (AUC) was 0.707 (Fig. 3).

### The expression of S100A4 in lung tissues and alveolar macrophages of mice with silicosis

**Fig. 4:**
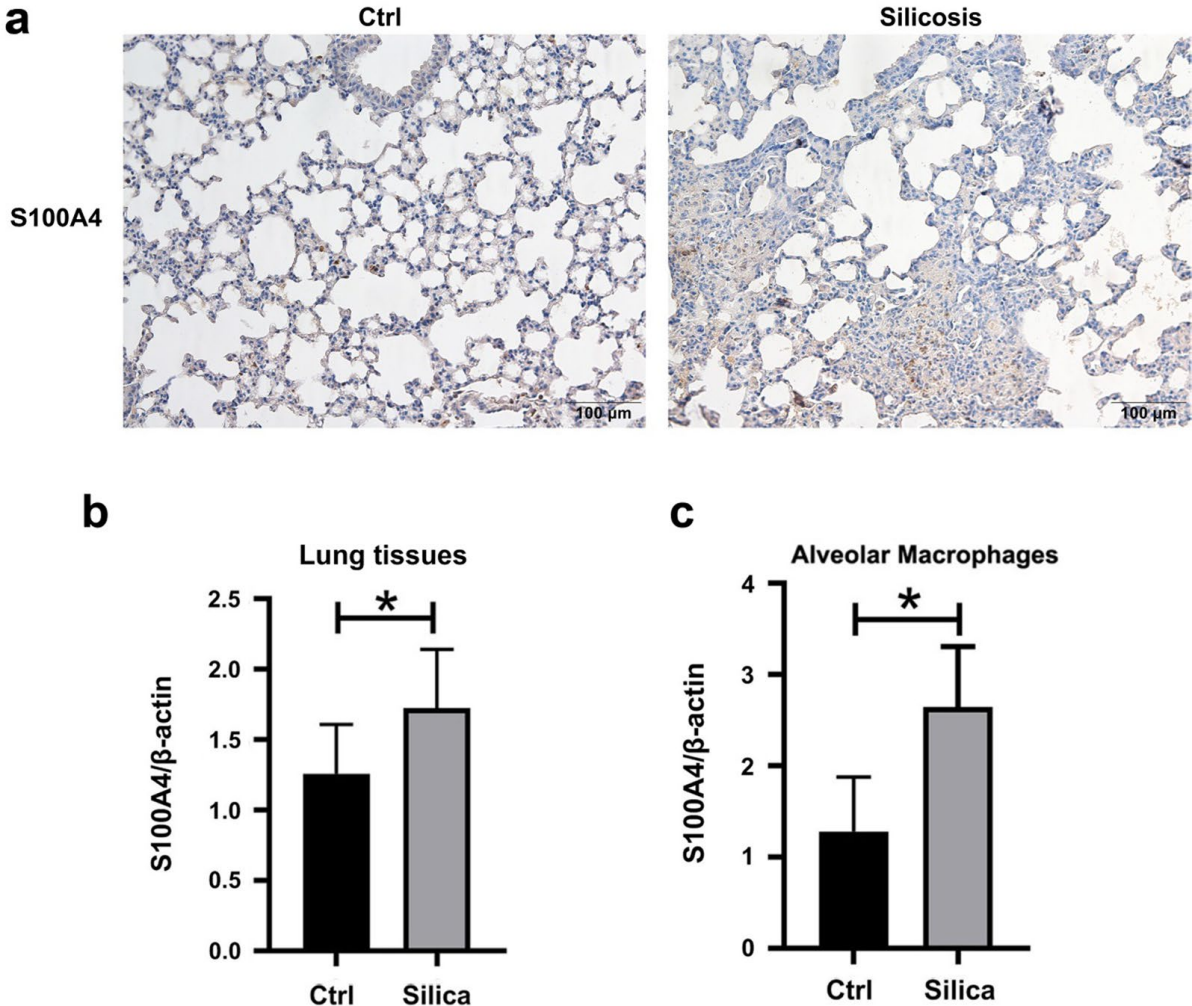
The expression of S100A4 in mice. The staining (**a**) and mRNA expression (**b**, **c**) of S100A4 in mice were detected by IHC and real-time PCR respectively, *p < 0.05

The staining of S100A4 was detected by IHC in the lungs of mice. Sparse expression of S100A4 was found in alveolar epithelial cells of control mice, while abundant S100A4-expressing cells were observed in the areas of nodules of silicotic mice (Fig. 4a). The mRNA expressions of S100A4 in the lungs of silicotic mice were significantly increased in comparison to controls (Fig. 4b). Furthermore, the alveolar macrophages in mice with silicosis exhibited higher expression of S100A4 compared with the control mice (Fig. 4c).

## Discussion

It is widely accepted that S100A4 plays a crucial role in the pathogenesis of fibrotic diseases. It has been found that S100A4 levels were significantly increased in the serum and BALF of IPF patients [18, 21]. Similar to these studies, we found that the levels of S100A4 were increased in the serum of patients with silicosis and in the lung tissues of silicotic mice. AKIYAMA et al. reported that most S100A4 + cells were accumulated around fibrotic areas in the lung tissues of IPF patients [21]. Moreover, the numbers of S100A4-positive macrophages were correlated with S100A4 levels in BALF of IPF patients and S100A4-positive macrophages were main source for extracellular S100A4 in the inflammatory phase of bleomycin-induced pulmonary fibrosis [18]. Then, our study also found that S100A4 was mainly expressed at the cellular nodules of silicotic mice, and primary AMs of silicotic mice exhibited a higher S100A4 level compared to control mice, suggested that AMs may be an important source of S100A4 in silicosis. S100A4 promoted the proliferation of lung fibroblasts and also drive myofibroblast differentiation by inducing the expression of the α-SMA and collagen I [18, 19], suggesting a critical role of S100A4 in deposition of collagen and profusion of fibroblastic foci in pulmonary fibrosis. Furthermore, our previous study found that the expression of S100A4 was increased in TGF-β1-induced epithelial-mesenchymal transition (EMT) in A549 and RLE-6TN cells. According to these findings, we speculate that elevated S100A4 may mainly origin from alveolar macrophages after engulfing silica in the inflammatory stage and contribute to fibrosis in silicosis by promoting the transition of fibroblasts and alveolar epithelial cells to myofibroblasts. This hypothesis can be supported by the findings in our study that serum S100A4 levels were positively correlated with TGF-β1 and IL-6, suggested a possible role of S100A4 in inflammation and fibrosis of silicosis. However, the detailed mechanism of S100A4 in silicosis needs further investigation.

Silicosis is characterized by sustaining inflammatory responses and progressive pulmonary fibrosis [8]. Inhalation of crystalline silica could activate immune and non-immune cells, such as epithelial cells, fibroblasts and macrophages, which could release inflammatory cytokines and fibrotic cytokines, such as TGF-β1, CTGF, TNF-α and IL-6 [5, 7, 25, 26]. Studies found that TNF-α and IL-6 were increased in the serum [27] and BALF [28] of patients with silicosis. TGF-β1 and CTGF, two classical pro-fibrotic cytokines, were also found to increase in serum of patients with silicosis and lung tissues of silicotic rat [29–31]. Similarly, our study found that the secretion of serum TGF-β1, CTGF, TNF-α and IL-6 were higher in patients with silicosis than in healthy subjects. We also investigated the levels of cytokines in stage I, II, and III of silicosis respectively. Specifically, the levels of TGF-β1, TNF-α and IL-6 in SII group were higher than those in CG group. Although the secretion of CTGF in SI, SII and SIII group were higher than in CG group, there was no significant difference between groups. The smaller sample size involved in the present study might be an important factor contributing to the inconsistent results obtained from our work and the former works.

Persistent inflammation of the alveoli and further pulmonary fibrosis can result in irreversible ventilatory impairment in silicosis [32–34]. FEV1, FVC and PEF are important parameters to evaluate airway obstruction of workers with silica exposure [3, 32, 35]. VC and IC are another two indexs to indicate lung capacity of patients. The present study assessed the lung function of individuals to estimate the association between silicosis and lung function. Consistent with previous studies [28, 36, 37], we found that FVC%pre, FEV1%pre and FEV1/FVC were significantly decreased in patients with silicosis. Moreover, Huang et al. found that sputum S100A4 levels in asthma patients were negatively correlated with FEV1, FEV1%pre and FEV1/FVC [38]. S100A4-positive basal epithelial cells in the airway of chronic obstructive pulmonary disease patients were inversely correlated with FEV1/FVC [39]. These two findings suggested a link between S100A4 and lung function in pulmonary diseases. Similar to these observations, we also found that S100A4 was negatively correlated with lung function parameter FVC%pre, PEF75, Vcmax and IC in the present study. Whether oversecretion of S100A4 impairs lung function or lung function impairment evokes the hypersecretion of S100A4 needs further study.

Several limitations of this study need to be discussed. First, the sample size was small in groups, especially in control group. Second, we only observed the association between S100A4 and silicosis, but detail molecular mechanisms are still unknown. Third, little is known about the value of S100A4 in clinical practice. Hence, future studies should enlarge the sample size to explore the clinical significance of S100A4 in early diagnosis of silicosis. And further mechanism of S100A4 in silicosis should be investigated in vivo and in vitro.

## Conclusions

Collectively, we found that S100A4 levels were increased in the serum of silicosis patients and the alveolar macrophages and lung tissues of silicosis mice. The secretion of S100A4 was correlated with inflammation, fibrosis and lung function in silicosis. The role of S100A4 as a potential therapeutic target for silicosis needs to be evaluated in further studies.

## Data Availability

The datasets analyzed during the current study are available from the corresponding author on reasonable request.

## Abbreviations

S100A4: S100 calcium-binding protein A4
EMT: Epithelial-mesenchymal transition
CG: Control group
TGF-β1: Transforming growth factor-β1
CTGF: Connective tissue growth factor
IL-6: Interleukin-6
TNF-α: Tumour necrosis factor-α
ROC: Receiver operating characteristic
AUC: Area under the curve
BALF: Bronchoalveolar lavage fluid
IPF: Idiopathic pulmonary fibrosis
BMI: Body mass index
FVC: Forced volume capacity
FEV1: Forced expiratory volume in 1 s
FVC%pre: Percentage of predicted forced vital capacity
FEV1%pre: Percentage of predicted forced expiratory volume in 1 s
FEV1//FVC: Ratio of forced expiratory volume in one second to forced vital capacity
Vcmax: Maximum vital capacity
IC: Deep inspiratory capacity
PEF: Peak expiratory flow
PEF25: Peak expiratory flow at 25% of vital capacity
PEF50: Peak expiratory flow at 50% of vital capacity
PEF75: Peak expiratory flow at 75% of vital capacity
